# Supplementary figures and images for: Comparison of Growth Performance and Meat Quality Traits of Commercial Cross-Bred Pigs versus the Large Black Pig Breed
Source: Animals (Basel). 2021 Jan 15;11(1):200. doi: 10.3390/ani11010200 (PMC7830199; doi:10.3390/ani11010200)

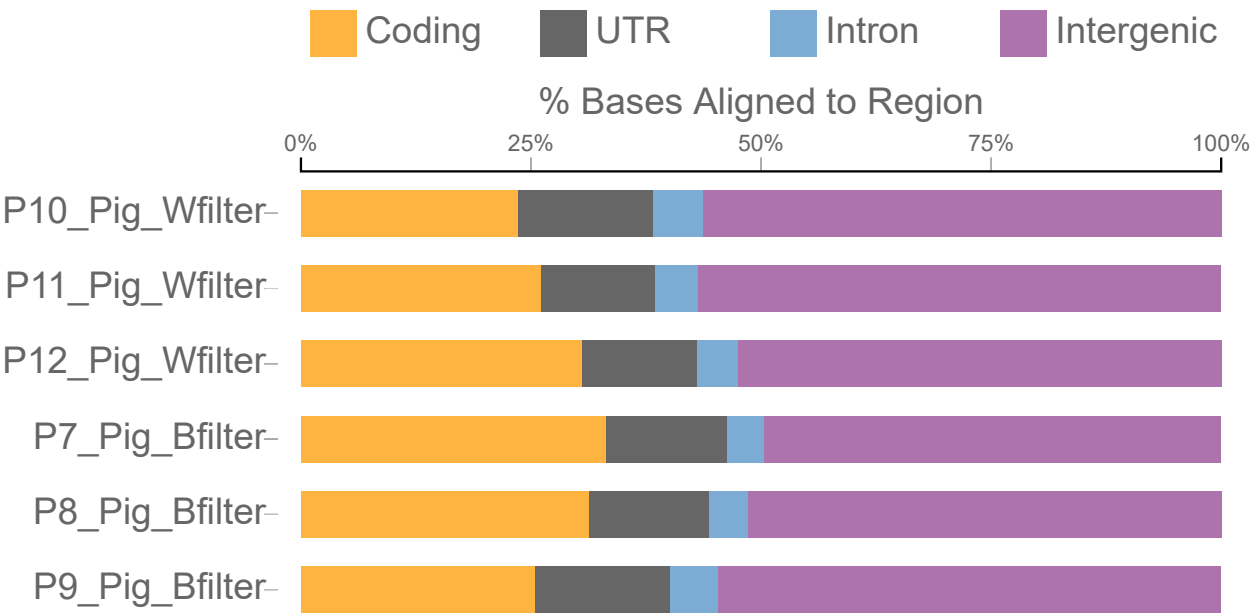

Supplement: Supplementary file 1 [file animals-11-00200-s001.zip › Figure S2 AlignmentDistributionPlots.pdf]
